# Constitutive MHC class I molecules negatively regulate TLR-triggered inflammatory responses via the Fps–SHP-2 pathway

**DOI:** 10.1186/1479-5876-10-S3-P7

**Published:** 2012-11-28

**Authors:** Sheng Xu, Jin Hou, Xingguang Liu, Chaofeng Han, Peng Zhang, Xuetao Cao

**Affiliations:** 1 National Key Laboratory of Medical Immunology & Institute of Immunology, Second Military Medical University, Shanghai, China; 2 National Key Laboratory of Molecular Biology, Chinese Academy of Medical Sciences, Beijing, China

## 

Toll-like receptors (TLRs) are key pattern-recognition receptors used by cells of the innate immune system to detect conserved components of pathogens, and have critical roles in host defense against microbial pathogens. However, the molecular mechanisms that fine-tune TLR-triggered innate responses remain to be fully elucidated. Previous studies suggested that major histocompatibility complex (MHC) molecules can mediate reverse signaling and have nonclassical functions[1,2]. The aggregation of MHC class I on cell surface activates signal pathways in T cells, B cells, tumor cells or endothelial cells and elicits various biological effects, such as cell apoptosis, activation or proliferation [3,4]. Crosslinkage of MHC class I on human NK cells induces intracellular tyrosine phosphorylation and inhibits NK cell cytotoxicity[5]. Here we found that constitutively expressed membrane MHC class I molecules attenuated TLR-triggered innate inflammatory responses via reverse signaling. TLR ligands triggered more inflammatory cytokines production in MHC I-deficient mice and macrophages. And MHC I-deficient mice were more susceptive to both endotoxin sepsis and *E. coli* infection, but more resistant to *Listeria monocytogenes*. The intracellular domain of MHC class I molecules was phosphorylated by the kinase Src after TLR activation, then the tyrosine kinase Fps was recruited via its Src homology 2 domain to phosphorylated MHC class I molecules. This led to enhanced Fps activity and recruitment of the phosphatase SHP-2, which interact with TRAF6 and inhibit its ubiqutination, and finally resulted in suppressed TLR-triggered innate inflammatory responses. We further found that MHC class I molecules on B cells could also attenuate BCR signaling by suppressing Lyn activation. So, constitutive MHC class I molecules may be not only antigen presenting molecules, but also involved in fine-tune of both innate and adaptive immune responses.
